# A systematic review of the effect of sandplay therapy on social communication deficits in children with autism spectrum disorder

**DOI:** 10.3389/fped.2024.1454710

**Published:** 2024-10-11

**Authors:** Ren Yuxi, Jia Shuqi, Liu Cong, Li Shufan, Long Yueyu

**Affiliations:** School of Physical Education, Shanghai University of Sport, Shanghai, China

**Keywords:** autism spectrum disorder, child, sandplay therapy, social communication deficits, systematic review

## Abstract

**Objective:**

To explore the efficacy of sandplay therapy in intervening social communication deficits in children with autism spectrum disorders (ASD), and whether this efficacy is influenced by the age of the children and the dosage of sandplay therapy intervention.

**Methods:**

Following the PICOS principle, randomized controlled trials (RCTs) related to sandplay therapy for social communication deficits in ASD children were retrieved from seven databases: PubMed, WOS, The Cochrane Library, Embase, CNKI, Wanfang, and VIP, from the inception of each database to November 10, 2023. Two experimenters independently conducted study screening and excluded studies with concomitant diseases, incomplete data, unextractable data, and non-randomized controlled trials. The PEDro scale was used for methodological quality assessment, and the GRADEprofiler method was employed to evaluate the quality of evidence. Stata17 software was used for meta-analysis, subgroup analysis, sensitivity analysis, and publication bias testing. The standardized mean difference (SMD) and 95% confidence interval (CI) were used as the effect statistics.

**Results:**

A total of 12 RCTs (791 cases) were included. Sandplay therapy had a positive impact on the social communication deficits of ASD children [SMD = −1.42, 95%CI (−1.79, −1.04), *P* < 0.001]. Subgroup analysis revealed that sandplay therapy administered during the early school age (449 cases, SMD = −1.44, *P* < 0.05), for a duration of 22–28 weeks (208 cases, SMD = 1.69, *P* < 0.05), and with a frequency of once per week (218 cases, SMD = −1.67, *P* < 0.05) was most effective in improving on social communication deficits of ASD children.

**Discussion:**

The quality of evidence in this study was rated as high, with good methodological quality, including 12 studies with better quality and no detection of bias risk. The study had high heterogeneity, which was attributed to the measurement tools and intervention duration through subgroup analysis, with no inconsistency found. Additionally, no downgrade factors related to imprecision, publication bias, or indirectness were identified. In conclusion, sandplay therapy is an effective measure to improve social communication deficits in children with ASD, and current evidence recommends early intervention using an individual sandplay therapy or integrated sandplay therapy intervention program once a week for 22–28 weeks, which can serve as evidence-based clinical guidance.

**Systematic Review Registration:**

www.crd.york.ac.uk, identifier (CRD420234821750).

## 1 Introduction

Autism spectrum disorder (ASD), also known as autism, is a group of neurodevelopmental disorders that occur most often in early childhood (1). In recent years, the incidence of ASD has been on the rise globally (2). According to the latest data from the Centers for Disease Control and Prevention, the annual incidence of ASD is 2.76% (3). The social communication deficits, characterized by persistent social communication and interaction deficits, is a core symptom of ASD, which runs throughout the entire course of the disorder. Due to the lack of social interaction (4, 5), patients are often subjected to peer rejection and social isolation, which seriously affects their individual development and recovery (6–8). Therefore, how to intervene and improve the social communication deficits of ASD children has become an urgent problem to be solved.

The pathological mechanism of ASD is not clear, and there is no appropriate drug treatment (9). At present, conventional rehabilitation therapy primarily includes applied behavior analysis, structured education, interventions for interpersonal relationship development, key behavior training, early Denver intervention, among other methods (10). These treatments have a certain rehabilitation effect on patients; however, in the intervention process, patients not only need to go through difficult behavioral training, but also need a long physiological and psychological adaptation (11, 12). Sandplay therapy, a psychological treatment technique based on non-verbal intervention, is a widely influential method and technique of psychotherapy in the international arena, which has been gradually recognized and widely used in rehabilitation interventions for children with ASD by virtue of the advantages of high applicability, no side effects and ease of implementation (13). It is applied in the fields of education, social services, the military, women and children, and serves as an adjunctive treatment in departments such as psychiatric units in hospitals (14–18). In addition, a number of standardized systems and methods have been developed, such as Jones' sandplay Worldview Scale (19) Ramos & de Matta's Taxonomy of sandplay Pictures (20), and Grubbs’ Classification of Expressive Patterns in the sandplay (21), which provide systematic approaches to understanding and studying sandplay therapy. A sandtray and a set of miniatures apply sand, water, and sand tools to the creation of imagery in the context of the doctor-patient relationship and the sandtray's “space of freedom and protection” (22). A large number of studies have confirmed that sandplay therapy can be used by the therapist to play according to the particularity of the ASD individual (23), guide children through social interaction and emotional expression (24), and touch stimulates children's brain nerves to play a role (25), thus improving the level of social interaction of children with ASD.

In recent years, there have been more and more clinical studies on the intervention of sandplay therapy in patients with ASD (26, 27). Previous studies have confirmed that sandplay therapy has a rehabilitative effect on ASD patients (28). However, due to the differences in outcome indicators, intervention methods and research objectives, the study on the intervention effect of the core symptom of social communication deficits is still inconclusive, and the influence of factors such as intervention period and sandtable intervention “dose” on outcome indicators is lacking. Based on this, this paper intends to use the method of meta-analysis to explore whether sandplay therapy is effective in influencing social communication deficits in children with ASD, whether the intervention effect is related to the age of patients, and whether factors such as the mode, cycle and frequency of sandplay therapy intervention have an impact on outcome indicators. Through this study, we hope to find the best intervention time and intervention effect, aiming at providing more accurate evidence-based support for clinical practice.

## 2 Research data and methods

### 2.1 Research program and registration

This study was reported in accordance with the PRISMA2020 Guidelines (Preferred Reporting Program for Systematic Review and Meta-Analysis) to ensure research transparency (29). The research proposal has been registered with PROSPERO under the registration number CRD42023482175.

### 2.2 Literature retrieval

Published studies in PubMed, Web of Science, EMbase, The Cochrane Library, CNKI, Wanfang, and VIP were searched from the establishment of the database to November 10, 2023. The retrieval process was carried out independently by two researchers. If there was any difference, the third researcher would discuss it together until they reached a consensus.

### 2.3 Document inclusion criteria

The study was included in accordance with PICOS principles, as shown in Table 1.

**Table 1:** PICOS inclusion criteria.

PICOS	Inclusion criteria
Participants	Children younger than 18 years of age who have been diagnosed with ASD or meet the diagnostic criteria for ASD in either the Chinese Classification and Diagnostic Criteria of Mental Disorders 3rd Edition (CCMD-3) or the Diagnostic and Statistical Manual of Mental Disorders
Intervention	The control group was treated with sandplay therapy
Control	Conventional rehabilitation therapy, drug therapy, social behavior intervention, structured education combined with auditory integration training
Outcome	Outcome indicator or partial outcome indicator was social interaction; The measurement tools were Autism Behavior Checklist (ABC), Autism Treatment Assessment Scale (ATEC),Social Responsiveness Scale (SRS), the higher the score of the above scale, the more serious the disorder
Study design	Randomized Controlled Trial

### 2.4 Document exclusion criteria

Studies were excluded if they met the following exclusion criteria: (1) Accompanied by visual and auditory impairment, physical diseases, other mental diseases, etc.; (2) The literature data is incomplete or cannot be extracted; (3) Reviews, case studies, qualitative studies, non-intervention studies, conference papers; (4) Non-randomized controlled trials; (5) The intervention is not a sandplay therapy.

### 2.5 Data extraction

Two researchers independently screened the study, extracted the data, calculated the data, and cross-checked according to the exclusion criteria. If there were differences, they discussed them or referred to the opinions of the third researcher until a consensus was reached. The researchers extracted the required data according to the pre-determined data, including the basic characteristics of the study (author, publication year, country), basic information of the subjects (sample size, age), intervention measures (detailed intervention measures of the experimental group and the control group), intervention dose (intervention time, intervention frequency, intervention duration), and main outcome indicators.

### 2.6 Quality evaluation

Methodological quality was evaluated using Physical Therapy Evidence Database (PEDro) scale (30). The scale included 10 items, including “random assignment”, “assignment hiding”, “baseline similarity”, “study object blinding”, “therapist blinding”, “outcome assessment blinding”, “participation rate >85%”, “intention-to-treat analysis”, “analysis of statistical results between groups”, and “point measurement difference value”. Each study can earn up to a total score of 10 points. Each scale item scores a standard with 1 point if the standard is met or 0 points if the standard is not met, with <4 being poor quality, 4–5 being moderate quality, 6–8 being better quality, and 9–10 being high quality; only studies of moderate quality or higher were included in this paper.

### 2.7 Evidence quality assessment

GRADEprofiler evidence grading system is used to evaluate the evidence quality of outcome indicators. The evaluation content of outcome indicators' evidence quality includes 5 degradation factors of publication bias, inconsistency, inaccuracy, indirectness and bias risk. The evidence grade is divided into four levels: high (not degraded), medium (degraded by 1 level), low (degraded by 2 levels), and very low (degraded by 3 levels) (31). The quality rating was conducted independently by two researchers. If there were any differences, the third researcher would discuss them together until they reached a consensus.

### 2.8 Statistical method

Stata17.0 software was used to perform effect size combination, subgroup analysis, sensitivity analysis and publication bias test. Effect indicators were calculated using standard mean difference (SMD). According to the Cohen effect size standard, 0.2, 0.5 and 0.8 were the boundary values of small, medium and large effect sizes, respectively (32). Higgins' I^2^ statistic was used for heterogeneity, with 75%, 50% and 25% as the boundaries of high, medium and low heterogeneity, respectively (33), If there is heterogeneity in the study, random effects model is adopted, and the source of heterogeneity is explored through subgroup analysis or sensitivity analysis. Publication bias is determined by Egger test, and 95% confidence interval is used as the result index of meta-analysis.

## 3 Results

### 3.1 Literature search results

**Figure 1:**
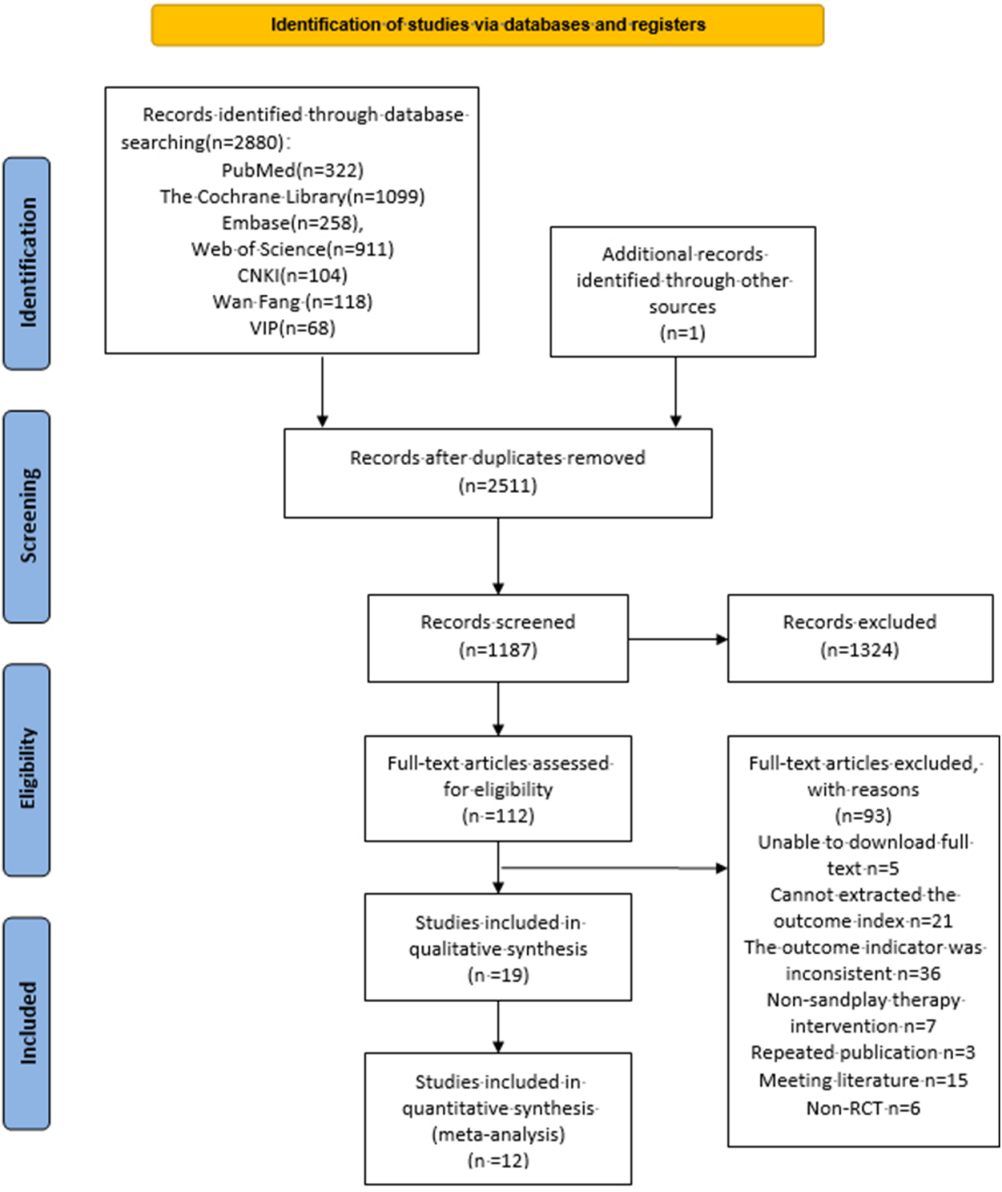
Literature retrieval process.

By searching seven databases, including PubMed, WOS, The Cochrane Library, Embase, CNKI, Wanfang and VIP, and referring to previous studies to supplement one paper, a total of 2,881 studies were retrieved. The study was imported into Endnote X9, and after de-weighting, 2,511 studies were obtained, 1,187 were obtained by reading the initial screening of titles and abstracts, and 93 were excluded after reading the full text, of which 5 could not be downloaded in full text, 21 could not be extracted from the endpoint indicators, 36 did not match with the endpoint indicators, 7 did not match with the intervention method, 3 were duplicated, 15 were conference studies, and 6 were non-randomized controlled experiments, and 12 were finally included (27, 34–44). The specific literature screening process is shown in Figure 1.

### 3.2 Basic features of the included literature

A total of 12 studies (791 individuals) were included, all of which were randomized controlled trials. Most of these trials were conducted in hospital settings and executed by professional sandplay therapists. The subjects were primarily children with ASD, aged 3–12 years. The sandplay therapy intervention modes mainly included two types: individual sandplay therapy (75%) and integrated sandplay therapy (25%). Individual sandplay therapy involves one-on-one intervention for children with ASD, with the process roughly as follows: doctor-patient trust building, guiding the creation of the work, and analyzing the work. Integrated sandplay therapy is based on individual sandplay therapy but includes age-appropriate children with normal socialization; the process is roughly as follows: guiding the establishment of social relationships, guiding the creation of the work, sharing the work, and archiving and dismantling the work. The control group received conventional rehabilitation, medication, social behavioral interventions, and a structured educational approach combined with auditory integration training. By staying that the frequency of interventions ranged from 1 to 7 times per week for 12–28 weeks, with each session lasting 45–60 min. None of the included studies reported adverse events; see Tables 2, 3.

**Table 2:** Basic characteristics included in the study table (*n* = 13).

Study	Country	Sample	Age (years)		Intervention characteristics	
		(E/C)	(E/C)		(E/C)	
Zhao et al. (43)	China	82/81	4.25 ± 1.09	4.35 ± 1.03	Ⅰ+a	a
Wu et al. (41)	China	45/35	6.00 ± 1.30	6.20 ± 1.20	Ⅱ+d	d
Sha et al. (40)	China	28/28	5.89 ± 1.23	6.03 ± 1.34	Ⅰ+c	c
Liu et al. (39)	China	25/25	NR	NR	Ⅱ+d	d
Li et al. (38)	China	22/22	NR	NR	Ⅱ+d	d
Guo and Li (27)	China	45/45	5.28 ± 0.81	5.36 ± 0.74	Ⅰ+a	a
Liu et al. (34)	China	26/26	4.50 (4.00–5.83)	4.90 (4.28–5.80)	Ⅰ+c	c
Hu (37)	China	52/52	5.76 ± 1.12	5.39 ± 1.75	Ⅰ+a	a
Cui and Ye (36)	China	4/4	7.00 ± 3.24	4.00 ± 5.57	Ⅰ+a	a
Chen and Chen (35)	China	12/12	5.44 ± 0.78	5.55 ± 0.91	Ⅰ+a	a
Zhang (42)	China	30/30	5.20 ± 1.05	4.90 ± 0.75	Ⅰ+a,b	a,b
Zhou et al. (44)	China	30/30	4.70 ± 1.60	4.30 ± 1.30	Ⅰ+a	a

**Table 3:** Table of intervention characteristics included in the study (*n* = 13).

Study	Intervention characteristics			
	Cycle (week)	Frequency (times/week)	Duration (min)	Outcome indicators
Zhao et al. (43)	12	NR	NR	ABC
Wu et al. (41)	24	2–3	45–60	ABC
Sha et al. (40)	18	2	50	SRS
Liu et al. (39)	12	2–3	45–60	ABC
Li et al. (38)	24	1	45–60	SRS
Guo and Li (27)	12	1	60	ABC
Liu et al. (34)	20	NR	45–60	SRS
Hu (37)	16	7	60	ABC
Cui and Ye (36)	12	NR	60	ATEC
Chen and Chen (35)	24	1	60	ATEC
Zhang (42)	24	1	60	ATEC
Zhou et al. (44)	12	NR	NR	ATEC

For the convenience of table making, only the first author is registered, NR indicates no registration, E experimental group, C comparator group, Ⅰ individual sandplay therapy, Ⅱ integrated sandplay therapy, a routine rehabilitation therapy, b routine medication therapy, c routine social behavior intervention, d structured education combined with auditory integration training, ABC, autism behavior checklist; ATEC, autism treatment assessment scale; SRS, social responsiveness scale.

### 3.3 Methodological quality of the included studies

The 12 randomized controlled trials included all met the “baseline similarity”, “intention-to-treat analysis”, “between-group statistical outcome analysis”, and “boint measurement and variance value”, as shown in Table 4. Among them, 12 studies achieved “random allocation” (27, 34–44), one study achieved “allocation concealment” (34), and three studies used “blinding of subjects” (35, 40, 42), one study achieved “blinding of outcome assessment” (40), and there was no “participation rate ≤15%”. In terms of the PEDro score, 12 studies scored 6–8 (27, 34–44), with a mean score of 6.4, and no low-quality study was seen, so the methodological quality was evaluated well.

**Table 4:** Methodological quality of the included studies.

Study	Random allocation	Allocation concealment	Baseline similarity	Blinding of subjects	Blinding of therapists	Blinding of outcome assessment	Exit rate <15%	ITT Intention to Treat Analysis	Statistical analysis between groups	Point measurement and variance value	Total score
Zhao et al. (43)	1	0	1	0	0	0	1	1	1	1	6
Wu et al. (41)	1	0	1	0	0	0	1	1	1	1	6
Sha et al. (40)	1	0	1	1	0	1	1	1	1	1	8
Liu et al. (39)	1	0	1	0	0	0	1	1	1	1	6
Li et al. (38)	1	0	1	0	0	0	1	1	1	1	6
Guo and Li (27)	1	0	1	0	0	0	1	1	1	1	6
Liu et al. (34)	1	1	1	0	0	0	1	1	1	1	7
Hu (37)	1	0	1	0	0	0	1	1	1	1	6
Cui and Ye (36)	1	0	1	0	0	0	1	1	1	1	6
Chen and Chen (35)	1	0	1	1	0	0	1	1	1	1	7
Zhang (42)	1	0	1	1	0	0	1	1	1	1	7
Zhou et al. (44)	1	0	1	0	0	0	1	1	1	1	6

### 3.4 Results of meta-analysis

**Figure 2:**
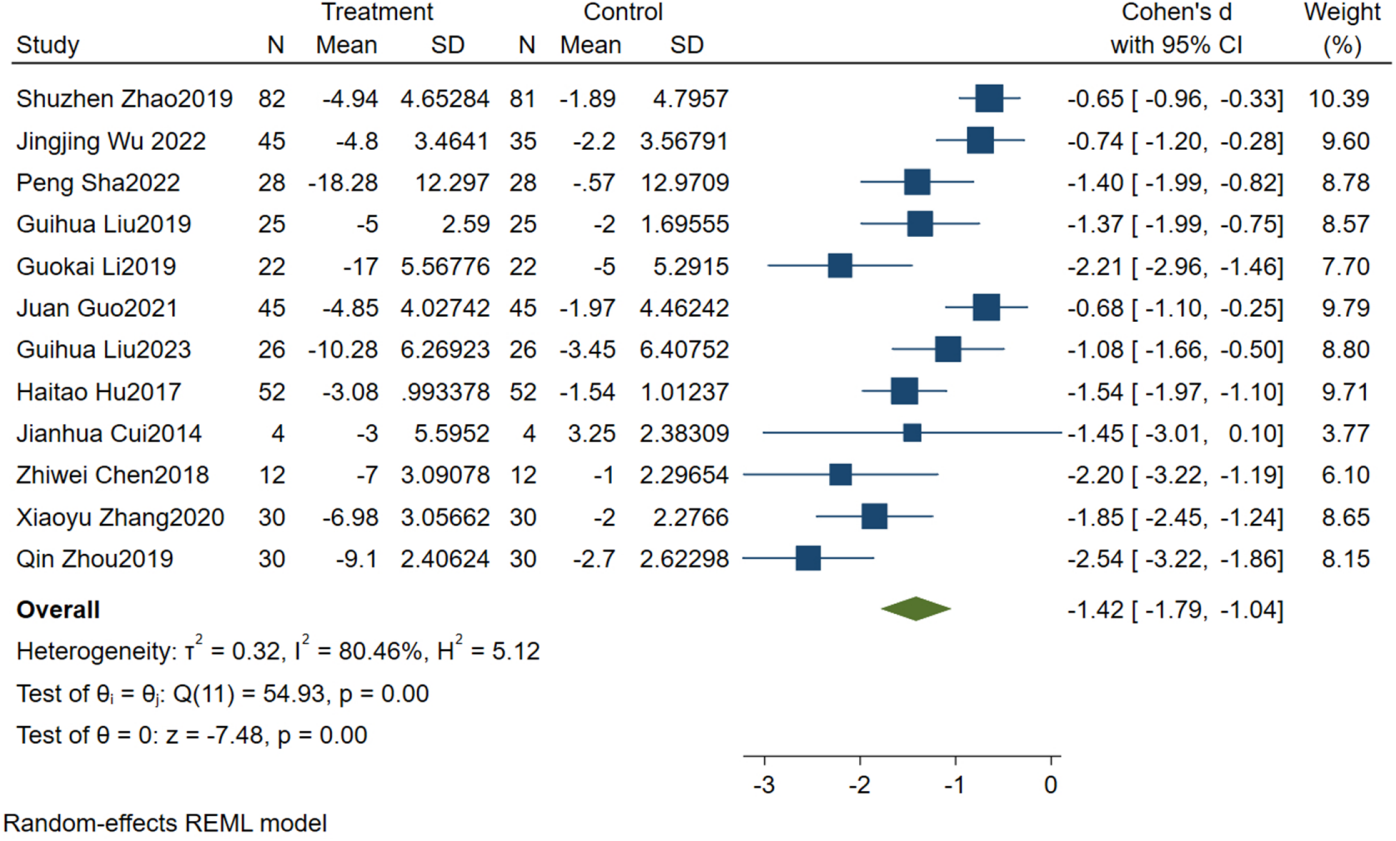
Forest diagram of the effect of sandplay therapy on social communication deficits in children with ASD.

A total of 12 studies were included, and the combined effect size was SMD = −1.42, 95% CI (−1.79, −1.04), *P* < 0.001, the difference was statistically significant, indicating that sandplay therapy can effectively improve social communication deficits in children with ASD. As shown in Figure 2, the heterogeneity test results showed that there was heterogeneity among the included studies (I^2^ = 80.46%). The random effects model was used to combine the data.

### 3.5 Sensitivity analysis

**Figure 3:**
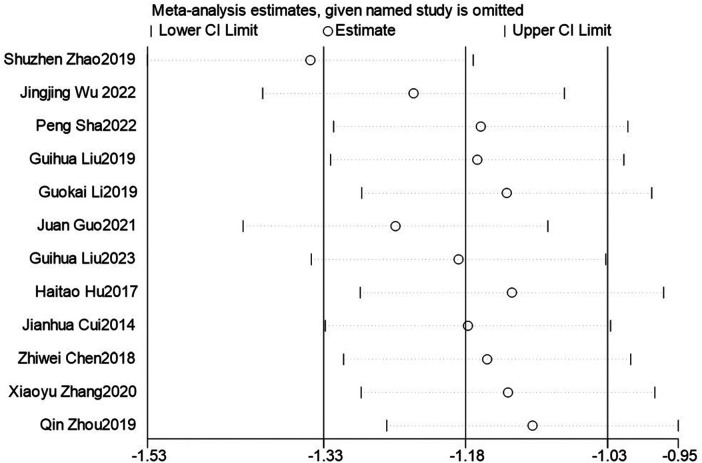
Sensitivity analysis.

In order to explore the source of heterogeneity, sensitivity analysis was performed using stata17.0. As shown in Figure 3, the combined effect is analyzed by eliminating individual studies one by one. After removing single studies, the range of SMD (−1.56 to −1.38) and I^2^ (75.67%–82.70%) were both <0.001, and no significant change was seen before and after removal. It shows that the sensitivity of the study data is low, and the results have certain reliability and stability.

### 3.6 Subgroup analysis

In order to further explore the source of heterogeneity, 5 moderating variables—measurement tools, intervention cycle, intervention frequency, sandplay therapy type, and patient age—were used to form subgroups. Other moderating variables had similar characteristics across studies, so no subgroups were created, as shown in Table 5. The results suggest that measurement tools and intervention cycle may be sources of heterogeneity.

**Table 5:** Subgroup analysis of the effect of sandplay therapy on social communication deficits in children with ASD.

Moderating variable		Research quantum	Heterogeneity test result			Results of meta-analysis		
			Q	*P*	I^2^	SMD	95% CI	*P*
Assessment tools	ABC	5	14.38	0.006	72.52	−0.97	(−1.338, −0.595)	<0.001
	ATEC	4	2.99	0.393	15.48	−2.12	(−2.565, −1.665)	<0.001
	SRS	3	5.54	0.063	65.57	−1.52	(−2.148, −0.900)	<0.001
Period/week	Long period (22–28)	4	16.98	0.001	79.07	−1.69	(−2.418, −0.955)	<0.001
	Medium period (15–21)	3	1.53	0.465	0.00	−1.38	(−1.678, −1.078)	<0.001
	Short period (8–14)	5	28.40	0.000	88.15	−1.29	(−2.018, −0.559)	0.001
Frequency (times/week)	1 time	4	19.99	0.000	81.52	−1.67	(−2.434, −0.906)	<0.001
	>1 time	4	6.81	0.078	54.94	−1.25	(−1.634, −0.865)	<0.001
The type of sandplay therapy	Individual sandplay therapy	9	43.77	0.000	81.77	−1.43	(−1.872, −0.986)	<0.001
	Integrated sandplay therapy	3	11.14	0.004	82.46	−1.40	(−2.224, −0.572)	0.001
Age of patients	Early School Children 3–5 years old	6	39.41	0.000	88.68	−1.44	(−2.090, −0.789)	<0.001
	School-aged children 6–12 years old	4	6.69	0.082	56.19	−1.24	(−1.691, −0.780)	<0.001

Age is divided according to Erickson's personality development stage in the early school age 3–5 years old, school age 6–12 years old.

Subgroup analysis divided the measurement tools for social communication deficits into three types: ABC, ATEC, and SRS, which were statistically significant (*P* < 0.05). Intervention cycles were categorized as short, medium, and long, with SMD values of −1.29, −1.38, and −1.69, respectively, showing statistical significance (*P* < 0.05). Intervention frequencies of once/week or more resulted in SMD values of −1.67 and −1.25, respectively, with statistical significance (*P* < 0.05). The types of sandplay therapy were categorized into individual and integrated sandplay therapy, with respective SMD values of −1.43 and −1.40, showing statistical significance (*P* < 0.05). When patients were aged 3–5 years and 6–12 years, the respective SMD values were −1.44 and −1.24, with statistical significance (*P* < 0.05).

### 3.7 Publication bias

**Figure 4:**
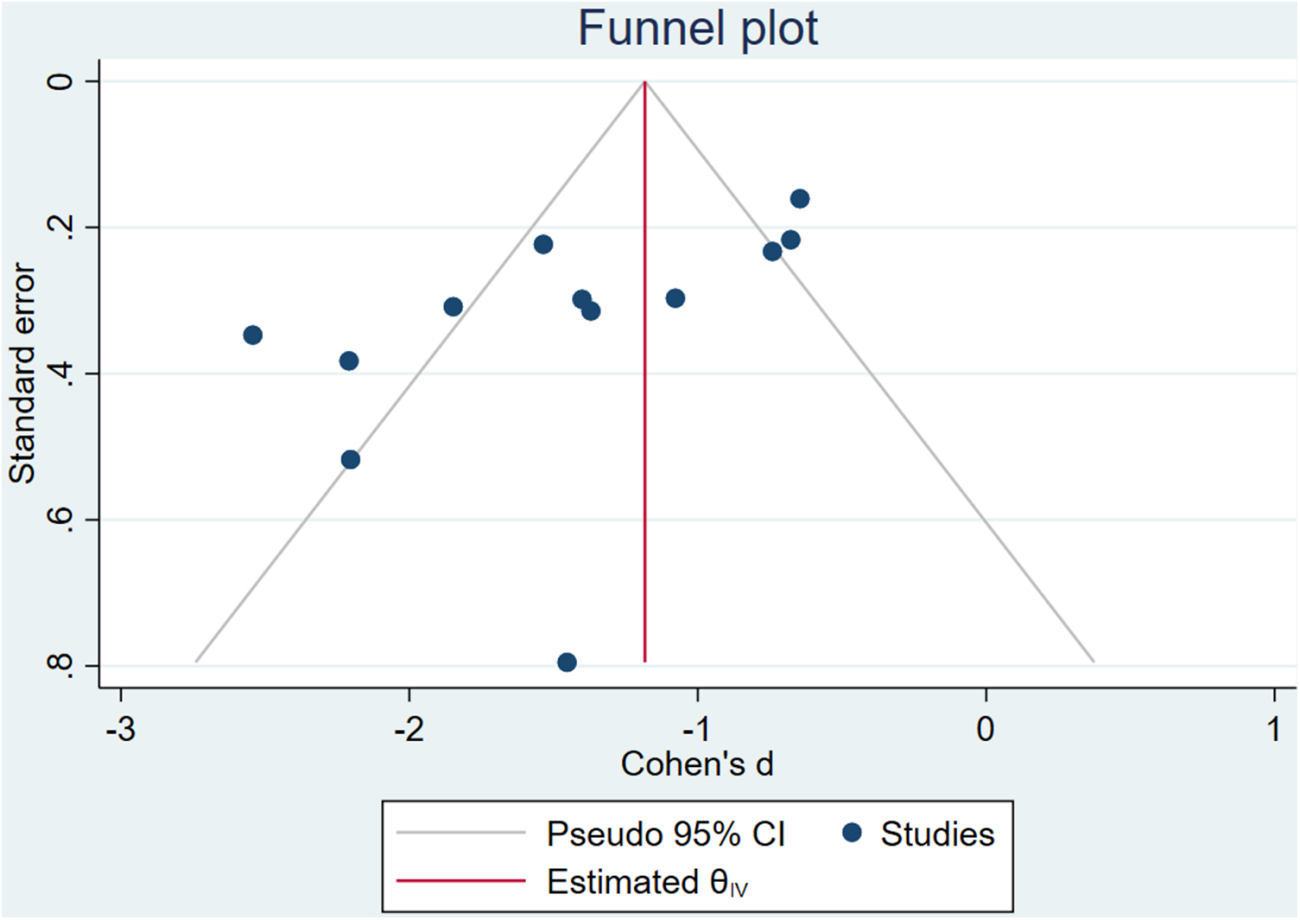
Egger's test results.

Egger test indicates that there may be potential publication bias, language bias and small sample study bias in this study (*Z* = −2.51, *P* > |t|=0.021 < 0.05). The publication bias analysis was conducted again after the clip-supplement method, and it was found that the effect size and confidence interval before and after the study did not change (SMD = −1.42, 95% CI: −1.786, −1.044) indicated that publication bias had little effect and the results were relatively robust, as shown in Figure 4.

### 3.8 Evidence quality assessment

**Figure 5:**
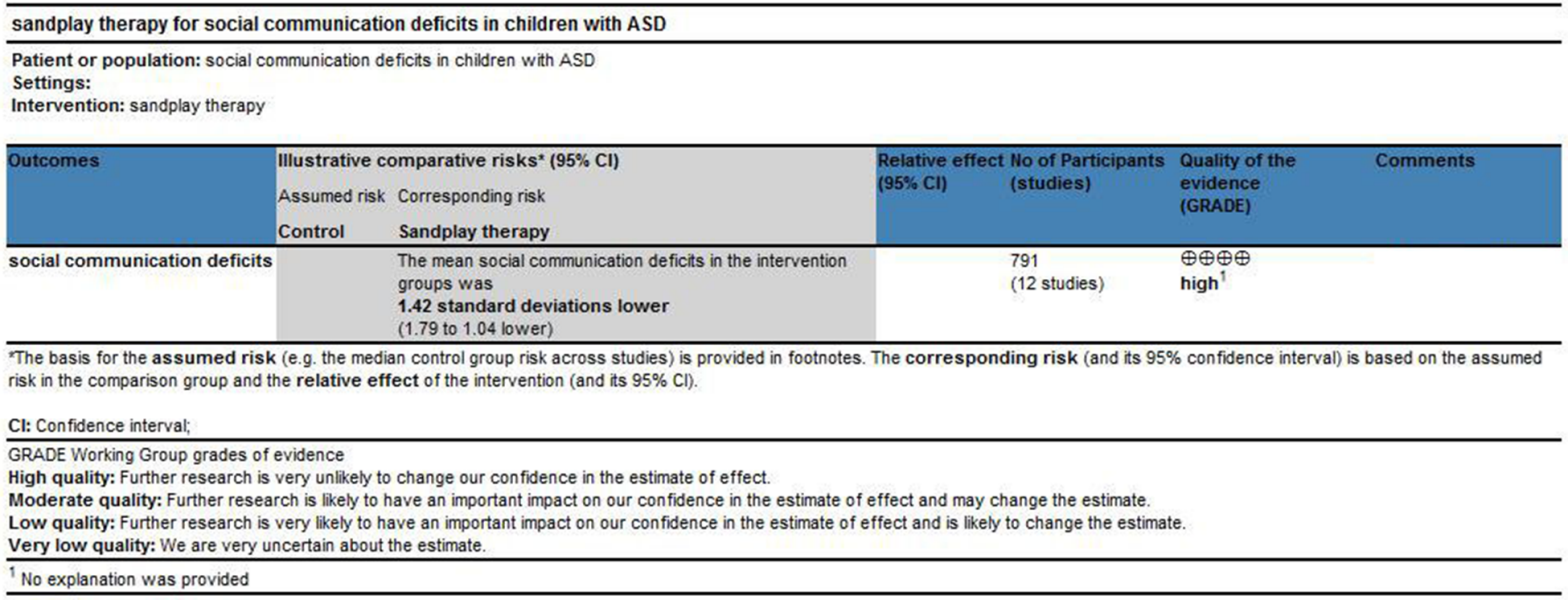
Evidence quality assessment.

GRADEprofiler evidence quality assessment shows that publication bias, inconsistencies, inaccuracies, inconsistencies, and risk of bias are not degraded. Sandplay therapy is rated as high in the quality of evidence assessment for improving social communication deficits in children with ASD, as shown in Figure 5.

## 4 Discussion

The results show that sandplay therapy has a significant effect on the improvement of social communication deficits in children with ASD, which is consistent with the results of previous reviews (45), sandplay therapy has also been found to have positive effects on social communication deficits in children with ADHD and hearing deficits (46, 47). Sandplay therapy enables patients to have more tactile opportunities, through which the peripheral nervous system transmits sensory information to the central nervous system (48), activates the cortical region and thalamus in the social brain network that process emotional touch (49), and causes the synthesis and release of oxytocin in the hypothalamus (50) thus enhancing the individual's ability to capture information, promoting social function (51).

A total of 12 studies (involving 791 cases) were included in this study to determine the intervention effect of sandplay therapy on social communication deficits in children with ASD. The PEDro scale was used to evaluate the study quality, yielding an average score of 6.4, with 12 studies rated as better quality. The overall quality of the studies was good, and no risk of bias was identified. Since I^2^ was greater than 75%, indicating high heterogeneity among studies, there may have been inconsistency. However, sensitivity analysis conducted one by one showed no significant changes, suggesting that the results are relatively stable. The heterogeneity was further explored due to differences in measurement tools and intervention durations. There were no downgrade factors related to imprecision, publication bias, or indirectness, so the quality of evidence in this paper was rated as high.

Sandplay therapy is an effective intervention for improving social communication deficits in children with ASD, and the measurement tool and intervention cycle in this study may provide a source of heterogeneity. Among the measurement tools, the ATEC has the most significant sensitivity (52, 53), and features detailed scale entries with high internal consistency in the assessment of social communication deficits in children with ASD. ATEC focuses on assessing treatment efficacy and severity of ASD-related symptoms (54), ABC focuses on observing and recording behavioral characteristics of children with ASD (55), and SRS focuses on social responses and communication skills (56). Some scholars have found that the sensitivity of the ATEC ranges from 0.922 to 0.987, which may confirm its validity and ability to capture changes in social skills (57). The higher the social-related scores on the above measurement tools the more severe their social communication deficits were, the intervention with sandplay therapy significantly improved, all with large effect sizes, and no adverse events occurred in all treatment groups. The diagnosis of ASD relies on the behavior of the ASD Core Symptom Assessment and is critical to the standardization of measurement tools. Sandplay therapy interventions of 12–28 weeks are effective for all children with ASD, with 22–28 weeks being more effective, and the complexity of the developing neurological system in children with ASD is influenced by genetic, epigenetic, and environmental factors (58), and there may be different consequences of impaired functioning for different children, and the intervention period may and does vary.

The results of the present study suggest that a weekly frequency of intervention may be more effective, possibly because the relationship between frequency and duration of intervention and treatment outcome is not simply linear (59). Weekly interventions may provide sufficient stability and continuity while avoiding overstimulation or fatigue. In terms of type of intervention, both individual sandplay therapy and integrated sandplay therapy have shown their effectiveness in the field of psychotherapy, where the therapist is required to continuously monitor and sensitively capture subtle changes in the child's behavior during the intervention. Among them, individual sandplay therapy with its one-on-one in-depth intervention model, the therapist is fully engaged in the interaction with the child with ASD (60) and accordingly makes immediate adjustments and optimization of the intervention strategy (61). This high degree of personalization and flexibility allows for precise matching to the specific needs and conditions of the child (62). As for integrated sandplay therapy, research suggests that although the mediating role of cognitive empathy in the interpersonal synchronization process of children with ASD is diminished compared to that of neurotypical children, it is not entirely lost (63). The integrated sandplay therapy can take advantage of this feature to stimulate children's cognitive empathy potential through game interactions, thus enhancing their interpersonal synchronization ability and laying a solid foundation for improving social skills.

In summary, sandplay therapy can be effective in improving social communication deficits in children with ASD, and the available evidence suggests an individual sandplay therapy or integrated sandplay therapy intervention type, with a frequency of one intervention per week for 22–28 weeks, at an early age. Sandplay therapy provides evidence-based practice guidelines for therapists and a strong scientific basis for clinical practice; The implementation of sandplay therapy encompasses multiple disciplines, including psychology, special education, and pediatrics, which has fostered the development of interdisciplinary research and clinical practice; Sandplay therapy offers a new intervention for children with ASD, which has immediate application for improving their social communication deficits; Sandplay therapy can be applied to children's mental health and personal growth, helping individuals to build self-confidence and a sense of self-worth, and is recommended to be promoted in family education, school education, and community services.

There are also limitations and future perspectives: (1) The sample of this study was mainly from China, and although there may be geographic and cultural variations in different countries, the diagnostic criteria for ASD in children tend to be more uniform, allowing for some degree of generalizability. There are more case studies in the available research and a lack of studies from different countries. In China, due to the high prevalence of children with ASD and the official establishment of the Chinese branch of the International sandplay therapy Society, the research and practice of sandplay therapy have significantly strengthened (64), but there is still the problem of small sample size. Future research should expand the sample size, increase the diversity of the population, use of more standardized measurement tools, and work to broaden its globalization in order to enhance the popularity of sandplay therapy. (2) The inclusion of subjects aged 3–12 years with limited information on spectrum characteristics and course of the disease may lead to potential differences in the effectiveness of interventions for children or adolescents of varying ages, spectrum characteristics, and course of the disease. The complexity of social skills increases with age and duration of illness, and social communication deficits in children with ASD may worsen in the future (65). Therefore the benefits of sandplay therapy need to be further explored.Future research could design more precise intervention programs tailored to the different ages, spectrum characteristics, and disease courses of children with ASD. (3) All of the included studies added sandplay therapy to the control group, but because of the variability in the interventions in the control group, there may be some impact on the efficacy of sandplay therapy to improve social communication deficits in children with ASD, and the harmonization of the interventions in the control group also poses a challenge for sandplay therapy. Future studies should aim to standardize the control group and increase the homogeneity of the articles to better explore the efficacy of sandplay therapy.

## 5 Conclusions

Sandplay therapy is an effective measure to improve social communication deficits in children with ASD, and current evidence recommends early intervention using an individual sandplay therapy or integrated sandplay therapy intervention program once a week for 22–28 weeks. Sandplay therapy, as a highly applicable, non-side-effective and easy-to-administer treatment, can effectively improve the social communication deficits of children with ASD, and may provide some clinical support for intervention strategies. Future research should also explore the intervention effect of sand play therapy on children who present across the broad autism spectrum, and develop more precise intervention programs, so as to help support children with ASD by improving their social and communication skills which may enhance their ability to engage meaningfully with peers and participate more fully in social contexts.

## Data Availability

The datasets used and/or analysed during the current study available from the corresponding author on reasonable request.
